# Therapeutics

**Published:** 1858-03

**Authors:** 


### THERAPEUTICS.

1. Experiments with Bibron's Antidote to the Poison of the Rattlesnake, etc. etc. *By William A. Hammond, M.D., Assistant Surgeon U.S. Army.*—Some four years since, Prince Paul, of Wurtemburg, the celebrated naturalist, communicated to my friend, Mr. De Vesey, the results of some experiments performed before the French Academy of Sciences by Professor Bibron, relative to an antidote to the poison of the rattlesnake. According to Prince Paul, Professor Bibron allowed a rattlesnake to bite him in the lips, cheeks, etc., and, by taking the antidote discovered by him, prevented all alarming symptoms, and, in fact, suffered no inconvenience therefrom.

The antidote in question, as stated by Prince Paul, is prepared according to the following recipe: ℞.—Potassii iodidi gr. iv; hydrarg. chloridi corros. gr. ij; bromini ʒv.—M. Ten drops of this mixture, diluted with a tablespoonful or two of wine or brandy, constitute a dose, to be repeated if necessary. It must be kept in glass-stoppered vials, well secured.

Prince Paul forwarded a small quantity of the above mixture to Mr. De Vesey, who used it successfully in the cases of two men bitten by rattlesnakes near his residence in Iowa.

During a recent expedition to the rocky mountains, I had several opportunities of testing its efficacy, and, since my return, have performed additional experiments with it. The results have been, upon the whole, exceedingly satisfactory, and I think that, when taken in time, it may be entirely depended upon in the poisonous wounds of the rattlesnake, and, perhaps, also in those of other venomous serpents.

1*st Experiment.*—Heinrich Brandt, acting hospital steward, was bitten, on the 2d of July, 1857, in the index finger of the right hand by a large rattlesnake, (*Crotalus confluentus,*) which he was in the act of putting into a jar for preservation. The snake inflicted a very deep wound, and hung by his fangs to the finger for a second or two before it could be detached. About four minutes after the bite, and before much pain or swelling had ensued, I administered one dose of Bibron's antidote. The symptoms almost immediately disappeared. Forty minutes after giving the first dose the pain and swelling returned, attended with considerable throbbing. I repeated the medicine, and in less than five minutes the finger had regained its natural appearance, and all pain and swelling had vanished. He remained perfectly well, and resumed his duties in an hour from the reception of the injury.

2*d Experiment.*—A very large rattlesnake was made to bite a young wolf (*Canis occidentalis*) about three months old. The serpent wounded the animal severely in the left flank. Fifteen minutes after the bite the leg was much swollen, and the wolf exhibited signs of great uneasiness, yawning, stretching, and looking about in an anxious manner. These symptoms continued to increase in intensity till inability to stand, drowsiness, and slight convulsive movements ensued. I now (thirty minutes from the infliction of the wound) gave six drops of the antidote, with the almost instantaneous disappearance of the observed symptoms. In a few minutes afterwards the animal ate a large piece of meat.

3*d Experiment.*—On the following day the same snake was made to bite the wolf three times in the space of five minutes, in the flank, neck, and chest. In two minutes after the last bite the effects of the poison were evidenced by the inability of the wolf to stand, gasping respiration, and a fixed expression of countenance. Some delay occurred in getting the antidote ready, and before I could administer it all signs of life had apparently ceased. Nevertheless, I placed six drops far down the throat, where it seemingly remained, as no effort of swallowing was perceived. However, in one minute respiration again commenced, and the heart could be felt to pulsate. The wolf lived for twenty-seven minutes, and then died comatose.

The rapidity of the action of the poison in this case, owing to the large quantity introduced into the system, prevented a successful issue. The good effects of the antidote were, however, sufficiently apparent to every observer, and I have no doubt that, had it been given before the faculty of swallowing was lost, the life of the animal would have been saved.

4*th Experiment.*—After my return to Fort Riley, a large *Crotalus confluentus*, which I had brought with me from the Rocky Mountains, was made to bite a dog five months old. The wound was made in the right shoulder. The poisonous effects of the bite commenced in ten minutes, causing gasping respiration, inability to stand, etc. I attempted to give a dose of the antidote, but the dog would not swallow, and I had no means at hand by which to introduce it into the stomach. I again tried to administer the remedy, but without success. The third dose was inhaled into the lungs. By this time the dog was perfectly senseless, and died in forty-five minutes after the infliction of the bite. Very slight swelling occurred in the wounded part.

5*th Experiment.*—Forty-five minutes after the last experiment the same snake was made to bite another dog of the same litter as the preceding. The wound was inflicted in the lower jaw, very near the mouth. At the end of three minutes, and before any violent symptoms ensued, a dose of the antidote was given. The dog swallowed it readily. Five minutes afterward the animal seemed very uneasy. Respiration was accelerated, and he preferred to lie down in the shade. At the end of about fifteen minutes he could stand with difficulty; and, as the sickness appeared to be on the increase, another dose was administered. Nearly half of this was lost. Slight swelling was now perceived in the face and neck. When roused the animal would walk a few yards, though with great difficulty, and evidently preferred rest and quiet. About one hour after the bite he lapped a little milk and seemed to be better, wagging his tail when spoken to, and walking with less effort. No increase of the symptoms occurred, and, in fact, the dog was, to all appearance, perfectly well in two hours after the reception of the injury, except that slight swelling of the under jaw still remained. I saw him no more till next morning, when this had disappeared and he was as active and lively as ever.

I had no further opportunities of repeating the experiments with other animals. During my absence, however, the antidote was used by Dr. Coolidge, U. S. Army, (to whom I am also indebted for assistance in the latter experiments,) in the following case, of which he has favored me with the subjoined account:—

"In July, 1857, a girl, aged fifteen years, was bitten, at Fort Riley, by a rattlesnake, on the dorsal aspect of the first phalanx of the ring finger of the right hand. In a few moments the finger became swollen and bluish, and when I first saw her, about ten minutes after the receipt of the wound, the forearm had begun to swell, and pain extended to the elbow. She was depressed and somewhat nauseated. An elder sister had sucked the wound from the first instant. There being sufficient space above the wound, I applied a cord tightly around the finger, and then made a free incision down to the bone. As soon as the articles could be procured from the hospital, I gave ten drops of the bromine mixture diluted, and injected into the wounded finger the preparation recommended by Dr. David Brainard, of Chicago, Illinois, (see *Annual Report Smithsonian Institution*, 1854.) viz.: ℞.—Iodinii, grs. x; Potasii iodidi, grs. xxx; Aquæ destillatæ, f℥j.—Solve. The patient expressed herself relieved after the first dose of the bromine; a second was given in twenty minutes. The solotion of iodine injected caused severe smarting pain; the fluid and air from the syringe could be felt a little above the wrist, and ultimately caused suppuration of the cellular tissue on the back of the hand. Nothing more was done. The girl recovered."

In conjunction with the mixture referred to in this paper, it will be observed that Dr. Coolidge laid open the wound and injected the cellular tissue with tincture of iodine, as recommended by Dr. Brainard, of Chicago, so that the favorable result in this instance cannot be attributed solely to the use of Bibron's antidote.—*American Journal of Medical Science*, January, 1858.

2. ON THE THERAPEUTICAL ACTION OF CHLORATE OF POTASH, WITH A NEW MODE OF ADMINISTERING IT. By *Dr. Dethan*.—Dr. Dethan considers that chlorate of potash is a powerful sialagogue, and that its elective action on the bucco-pharyngeal mucous membrane is well marked. To this physiological action is added a very remarkable and valuable success in pathology; its rapid and incontestible effects in mercurial salivation, by checking the formidable mercurial affection, have permitted practitioners to continue the mercury without fear, and thus to contend without remission against the constitutional infection. As an especial and incontestible remedy in ulcero-membranous stomatitis, this medicine need not, according to the physicians of the Hôpital Sainte-Eugénie, be swallowed; its topical application is sufficient, and in a short time the mucous membrane recovers its normal qualities and functions. Dr. Dethan concludes that the chlorate of potash, administered under a special form, which would permit the local action to be exercised slowly and certainly, although leaving the medicine to be carried into the stomach in a state of solution with the mixed liquids of the salivary, buccal, and pharyngeal glands, would be the mode of administration which would combine all indications and all opinions. He therefore suggests the use of the remedy in the form of pastiles, so that the patient may have at hand a remedy against the injurious effects of a mercurial treatment which he may be undergoing. The experiments of Dr. Ricord, and the publications of M. A. Fournier, testify incontestibly in favor of this successful simultaneous medication. In certain forms of angina attended with fibrinous exudations, it prevents the intimate adherence of the false membranes to the mucous membrane, and facilitates their expulsion, and assists the action of emetics. In this affection the topical action of the chlorate, favored by the bruising between the teeth, the natural solution in the liquids of the mouth, and its penetration into all the points interested, will be certainly efficacious. In debilitating diseases, such as diphtheritis, and gangene of the mouth, the child will find an agreeable and reparative kind of food, together with the most appropriate remedy hitherto discovered, against these diseases.—*British and Foreign Med.-Chir. Review*, January, 1858; from *L'Union Médicale*, June 4th, 1857.

